# Is There a “Right” Amount of Oxygen for Preterm Infant Stabilization at Birth?

**DOI:** 10.3389/fped.2019.00354

**Published:** 2019-09-06

**Authors:** Ju Lee Oei, Maximo Vento

**Affiliations:** ^1^Department of Newborn Care, The Royal Hospital for Women, Randwick, NSW, Australia; ^2^School of Women's and Children's Health, Faculty of Medicine, University of New South Wales, Randwick, NSW, Australia; ^3^Division of Neonatology, University and Polytechnic Hospital La Fe, Valencia, Spain

**Keywords:** oxygen, preterm infant, resuscitation, outcomes, review

## Abstract

The amount of oxygen given to preterm infants within the first few minutes of birth is one of the most contentious issues in modern neonatology. Just two decades ago, pure oxygen (FiO_2_ 1.0) was standard of care and oximetry monitoring was not routine. Due to concerns about oxidative stress and injury, clinicians rapidly adopted the practice of using less oxygen for the respiratory support of all infants, regardless of gestational maturity and pulmonary function. There is now evidence that initial starting fractional inspired oxygen may not be the only factor involved in providing optimum oxygenation and that the amount of oxygen given to babies within the first 10 min of life is a crucial factor in determining outcomes, including death and neurodevelopmental injury. In addition, evolving practice, such as non-invasive respiratory support and delayed cord clamping, need to be taken into consideration when considering oxygen delivery to preterm infants. This review will discuss evidence to date and address the major knowledge gaps that need to be answered in this pivotal aspect of neonatal practice.

## Introduction

The optimum amount of oxygen required for the respiratory support of newborn infants is one of the most contentious issues in current neonatal practice. The adverse effects of hypoxia are well-known and for centuries, pure oxygen was used without question (1). As recently as the 1960s, oxygen was considered “only to be good” and clinicians were advised to “use (it) liberally” (2), especially with knowledge that birth-related neurological injury could be related to hypoxia (3). The development of the Apgar Score in the 1950s encouraged oxygen treatment (4), even when Apgar herself showed that there was little relationship between birth oxygenation and later intelligence (5).

However, in the 1990s, the Resair study raised the possibility that room air (FiO_2_ 0.21) could be used instead of pure oxygen for newborn resuscitation (1). This study was conducted in India (6) where access to oxygen was limited and birth asphyxia rates were high. This study showed that air could be used as safely as oxygen to initiate the resuscitation of hypoxic full-term infants.

Over the next 15 years, an increasing number of studies (7–13) showed that air resuscitation was possible and that using air considerably reduced oxidative stress and injury to major organs such as the heart and kidneys (9). In 2005, a meta-analysis of >1,300 infants by Tan et al. provided compelling evidence that air resuscitation could decrease the risk of death in hypoxic infants by about 30% when compared to oxygen resuscitation (typical Odds Ratio (OR) 0.69, 95% Confidence Intervals (CI): 0.54–0.88; 14).

Thereafter, expert committee recommendations changed significantly. In 1998, pure oxygen was recommended as a supplement to infant ventilation support at birth (14). In 2006, guidelines suggested for the first time that air could be used for term infant resuscitation if oxygen was not available (15). In 2010, guidelines were once again revised to suggest that FiO_2_ be manipulated to target preductal SpO_2_ readings derived from healthy, full term infants (16–18). These recommendations have not changed to date (19).

Extremely preterm infants may not be hypoxic but still require respiratory support at birth. Many need supplemental oxygen to prevent hypoxia (20). The respiratory needs of preterm infants were acknowledged by international guidelines, which, over the years, varied from no specific recommendation (15), to “initiation of resuscitation with 30 or 90% oxygen and titration to oxygen saturation” (16), to recommending against use of high supplementary oxygen concentrations (65–100%) and advocating for the use of lower oxygen concentrations (21–30%) for infants below 35 weeks gestation (19).

However, whether lower oxygen strategies are best for preterm infants, considering the risk of pulmonary immaturity, is unknown. This review will therefore present current evidence to date for the use of oxygen in the delivery room respiratory support of preterm infants and discuss strategies to address knowledge gaps around contemporary practice. Specifically, it will delineate the historical context behind the shift in practice from higher to lower oxygen, evidence for short and long-term outcomes with lower oxygen resuscitation strategies and then the integration of evidence with current practice, including for more mature (e.g., >32 weeks gestation) preterm infants.

## Methods

This is an objective review of current literature and includes consideration of human and animal studies as well as clinical practice guidelines (CPGs).

### The History of Oxygen in Newborn Infant Resuscitation

For millennia, some newborn infants were acknowledged to need assistance to complete the necessary transition from intra to independent extra-uterine life. Chest expansion was the crucial step in this process and was usually accomplished with mouth to mouth by the accoucher (1). Oxygen was added after its discovery by Scheele and Priestley in the eighteenth century by the French anatomist, Francois Chaussier, who, in 1781, was the first to use oxygen to revive “apparently dead” newborn infants (1). Over the next two centuries, oxygen became an indispensable step in the newborn resuscitation process and was given in a wide variety of ways, including via the trachea, into the umbilical veins and even into the stomach [gastric mucosa was purported to have excellent oxygen absorbing properties (1)].

The role of oxygen for newborn resuscitation was firmly entrenched when hypoxia was linked to neurological injury in the 1940s (20). In 1957, the Apgar score was devised to systematically evaluate newborn resuscitation (4). One of the components of the Apgar score was “color.” Infants who were entirely “pink” received the maximum score of 2, those with blue extremities (acrocyanosis) received a score of 1 and those who were either white or blue, received a score of 0. Most infants became “pinker” with oxygen therapy, resulting in higher color and total scores. There was no apparent relationship between oxygenation at birth and later intellectual development (5) but despite this, the use of oxygen in the delivery room remained unquestioned for decades.

### Evolution of Oxygen Administration in the Delivery Room: When Less Became More

Whether a few minutes of exposure to pure oxygen at birth was safe was initially questioned by animal and bench studies (21). In the hypoxic milieu, high energy compounds such as adenosine triphosphate (ATP) are metabolized into hypoxanthine which in turn, is converted by the enzyme xanthine oxidase into xanthine and uric acid. Oxygen is a precursor for this reaction which produces reactive oxygen species (ROS), such as superoxide dismutase and hydrogen peroxide as by-products. The addition of oxygen, e.g., during resuscitation, increases the production of ROS, which, if unmitigated by anti-oxidant protection, eventually causes cellular damage and death (22).

In 1993, Ramji and his colleagues demonstrated that hypoxic full-term infants could be resuscitated with air instead of oxygen. This study was conducted in India, where access to oxygen was limited and costly. The Resair study randomized 84 asphyxiated babies to resuscitation at birth with either air or oxygen. Six infants in the air arm were given oxygen when they failed to respond (by increasing heart rate) within 90 s of life but was no difference in mortality rates or severity and incidence of encephalopathy between the two arms. An infant in the air arm was excluded because of lack of response to resuscitation efforts and was considered a still birth. The authors concluded that air was as safe as oxygen for hypoxic infant resuscitation (6).

Subsequent RCTs, totalling more than 1,500 infants, were then conducted over the next 15 years to examine this question (6–13). These studies led to a series of meta-analyses, with the latest conducted in 2018 (23–30), all of which concluded that air was unequivocally superior to oxygen in reducing the risk of death from birth asphyxia. The latest, conducted in 2018 by Welsford et al., noted that no new evidence had been acquired since 2007 and that the data from which the recommendations were based were low quality for the very important outcomes of mortality (Risk ratio [RR] = 0.73; 95% confidence interval [CI]: 0.57–0.94) and hypoxic ischemic encephalopathy (HIE, 5 RCTs; *n* = 1,315; RR = 0.89; 95% CI: 0.68–1.18). Furthermore, only one study has published on longer-term, i.e., post hospital discharge, outcomes. Saugstad et al. examined 213 of the 323 eligible infants enrolled in the Resair 2 study and found no difference between the air and oxygen groups in rates of cerebral palsy and neurodevelopmental delay (31).

### When Less Becomes a “Bit More:” the Implications of Transitional SpO_2_ Data

SpO_2_ is peripheral capillary oxygen saturation, the fraction of oxygen saturated hemoglobin relative to total (saturated and unsaturated) hemoglobin in blood. SpO_2_ is measured non-invasively by pulse oximetry, which, though not always identical to arterial oxygen saturations (SaO_2_), provides a safe, convenient, and inexpensive way of assessing oxygenation within the clinical environment. Normal adult pulse oximetry values range from 95 to 100% but in newborn infants, observational prospective cohort studies show that pre-ductal SpO_2_ in healthy term infants may take up to 14 min to minutes to reach ≥90% (17, 18, 32–34) and could be as low as 81% by 5 min in otherwise healthy infants delivered by cesarean section (34).

The implications of SpO_2_ targeting on infants with lung pathology are uncertain. None of the studies of HIE infants acquired SpO_2_ data in response to air resuscitation. Apgar measured oxygen content in 1,787 cord and heel blood samples from 404 infants between birth and 3 days of age. These infants were heterogenous, ranging from healthy infants to those with respiratory distress (*n* = 6), seizures (*n* = 7), and congenital problems, including “mongolism,” microcephaly and muscular dystrophy. There was no apparent relationship between blood oxygen content and IQ in 275 children that returned for developmental testing up to 4 years of age (5).

Nevertheless, recommendations to titrate FiO_2_ in response to SpO_2_ were made in 2010 (16), primarily from information derived from preterm infants below 30 weeks gestation. In this cohort, exposure to high (>80%) levels of oxygen at birth leads to rapid increase of SpO_2_, above 90%, by a few minutes of age (35, 36). In contrast, using air alone led to rapid decline of SpO_2_ (37), necessitating supplemental oxygen in almost all infants by 5 min of age to prevent hypoxia (36, 37) while adjusting FiO_2_ by graded increments emulated SpO_2_ trajectories of healthy term infants (38, 39).

### The Impact of a Few Minutes of Oxygenation on the Preterm Infant: the Important Outcomes

The preterm infant responds very differently to oxidative stress from the term infant. Despite lung protective and maturing therapies such as antenatal steroids (40) and exogenous surfactant (41), the preterm infant is exquisitely sensitive to oxidative stress as antioxidant defenses are not acquired sufficiently from the mother or produced *de-novo* until the 3rd trimester (42). The detrimental effects of chronic exposure to high concentrations of oxygen have been known for decades (43, 44) but the effects of hypoxia are equally serious. In an individual patient meta-analysis of five RCTs conducted between 2005 and 2014, enrolling 4,965 infants below 28 weeks gestation), Askie et al. found that nursing infants in lower SpO_2_ target ranges (85–89%) decreased the risk of retinopathy of prematurity (ROP) but increased the risk of death and necrotizing enterocolitis [NEC, (45)]. Nevertheless, the impact of just a few minutes of hyper or hypoxia in the preterm infant at birth is uncertain. The short period after birth is often overshadowed by the many events that occur after the infant is admitted into the NICU and a direct relationship between birth resuscitation and later outcomes is unclear.

### Can Preterm Infants Be “Resuscitated” With Less Than Pure Oxygen?

In 1989, Svenningsen et al. described the outcomes of 65 Swedish infants below 900 g (22–31 weeks) gestation born between 1984 and 1986. These infants were resuscitated with standard Swedish policy by ventilation with either “air or 30–40% oxygen by 1–5 min if the infant was not breathing and crying within the first minute after birth.” In this cohort, 52% of the infants survived at 28 days and 48% were alive at 1 year. The ontogeny of this policy is unclear but represents the feasibility of lower oxygen respiratory support in a high risk group of extremely preterm infants (46).

Based on this study, Lundstrom et al. randomly assigned a group of preterm infants (median gestation 29 weeks) in Denmark between 1991 and 1992 to resuscitation at birth with either air (FiO_2_ 0.21, *n* = 34) or FiO_2_ 0.8 (*n* = 38). This was the first RCT to describe both oxygen blending and SpO_2_ targeting in either term or preterm infants. In the air group, FiO_2_ was increased by steps of 0.1 steps if the “heart rate failed to normalize” within 1 min of age. FiO_2_ in the 0.8 group was not altered. Oximetry was determined in only 12 infants from each group because of equipment availability (35). Infants who were given FiO_2_ 0.8 had higher SpO_2_ readings (>90% by 3 min) compared to a control group of 12 healthy term infants while infants given air had SpO_2_ that approximated those of the term infants, reaching >90% only after 7–8 min of life. No infant died in the delivery room but those who were given FiO_2_ 0.8 had significantly lower cerebral blood flow, that was correlated with reduced survival in animal studies (47). No other outcomes, including neurodevelopmental outcomes, were described.

### The Evidence for Using Less Oxygen in Preterm Infants

To date, 11 RCTs (35, 37–39, 48–54) and four cohort studies (36, 55–57) have been conducted to determine the association between lower and higher oxygen strategies at delivery and preterm (<32 weeks) infant outcomes (see Table 1). All of these studies were conducted over a prolonged period of time (>10 years), with enrolment spanning between 1991 (35) and 2014 (54). As a consequence, the methodologies used by these studies were vastly different, especially for SpO_2_ targeting. Studies that recruited after publication of the 2010 guidelines (16) adjusted FiO_2_ to target SpO_2_ recommended by these guidelines (51–53). Older studies targeted SpO_2_ levels that were derived from best available evidence at the time of inception (35, 37–39, 48–50, 54). It is important to note that none of the RCTs were large enough to demonstrate any difference in the major outcomes of death and/or disability. Recruitment for the largest study, the To2rpido study (54), was curtailed at 15% of the target sample due to loss of equipoise against the 100% oxygen arm.

**Table 1:** Randomized controlled trials and cohort studies examining the use of lower and higher oxygen strategies in preterm infants.

	**FiO_**2**_**	***N***	**Gestation (weeks)**	**Death^#^**	BPD^**^	**PDA^#^**	**NEC^#^**	ROP^**^	**IVH^#^**
**RANDOMIZED CONTROLLED TRIALS**									
**Lundstrøm 1995, Enrolled 1991–1992, Denmark** **(****35****)**									
Low	0.21	34	29 (25–32)	2 (6)	5/32	4 (12)	2 (6)	1 (3)	2 (6)
High	0.8	35	29 (24–32)	6 (17)	2/29	7 (20)	1 (3)	2 (6)	3 (9)
**Harling 2005, Enrolment years not available, FiO**_****2****_ **not changed during resuscitation, no SpO**_****2****_ **monitoring, UK** **(****48**)									
Low	0.5	26	27 (23–31)	4	9/22	4	2	0	NR
High	1.0	26	28 (24–31)	5	7/22	2	0	0	NR
**Wang 2008, Enrolment 2005–2007, California, USA** **(****37**)									
Low	0.21	18	28.1 ± 2.2	1 (6)	7 (39)	4 (22)	1 (8)	1 (5)	1 (11)
High	1.0	23	27.6 ± 2.1	1 (4)	3 (13)	7 (38)	1 (9)	0	0
Escrig 2008* **Enrolment 2005–2007, death data collected at 28 days, Spain** **(****39**)									
Low	0.3	19	26.4 ± 1.9	4 (21)	4 (27)	10 (53)	0	1 (7)	2 (11)
High	0.9	23	26.1 ± 1.5	3 (13)	7 (35)	11 (48)	1 (4)	2 (10)	4 (17)
Vento 2009* **Enrolment 2007–2008, death data collected at 28 days, Spain** **(****38**)									
Low	0.3	37	26.1 ± 1.5	4 (11)	6 (18)	19 (51)	2 (5)	4 (12)	7 (19)
High	0.9	41	26.3 ± 1.3	3 (7)	13 (47)	27 (66)	1 (2)	6 (16)	5 (12)
Rabi 2011* **Enrolment between 2005 and 2007, Canada** **(****41**)									
Low	0.21	34	29 (28–30)^∧^	1	18/33	NR	NR	NR	NR
High	1.0	38	28 (28, 29)^∧^	1	22/37	NR	NR	NR	NR
**Armanian 2012, Enrolment 2009–2010, 29–34 weeks, Iran** **(****49**)									
Low	0.3	16	32	0	NR	NR	NR	NR	NR
High	1.0	16	30.8	0	NR	NR	NR	NR	NR
**Kapadia 2013, Enrolment between 2010 and 2011, USA** **(****51**)									
Low	0.21	44	30 ± 3	2 (4)	3 (7)	6 (14)	1 (2)	1 (2)	1 (2)
High	0.3	44	30 ± 3	3 (7)	11 (25)	10 (23)	6 (14)	4 (9)	1 (2)
Aguar 2013* **Enrolment between 2010 and 2012, Spain** **(****52**)									
Low	0.3	34	27.1 ± 1.6	4 (12)	10 (33)	23 (68)	2 (6)	4 (13)	11 (32)
High	0.6	26	26.7 ± 1.5	7 (27)	6 (32)	15 (58)	1 (4)	1 (5)	8 (31)
Rook 2013* **Enrolment between 2008 and 2012, Spain, Netherlands** **(****53**)									
Low	0.3	99	28.5 (27.1–30.3)^∧^	6 (6)	23 (24)	35 (35)	4 (4)	6 (6)	8 (8)
High	0.65	94	29.2 (26.3–30.4)^∧^	10 (11)	14 (17)	28 (30)	3 (3)	5 (5)	10 (11)
**Oei 2015 Enrolment between 2009 and 2014, Australia, Malaysia, Qatar** **(****54**)									
Low	0.21	144	28 ± 2	14 (10)	34 (24)	36 (25)	5 (4)	4 (3)	2 (1)
High	1.0	143	28 ± 2	6 (4)	40 (28)	41 (29)	1 (1)	8 (6)	6 (4)
**COHORT STUDIES**									
**Dawson Enrolment 2006–2007, Australia** **(****36**)									
Low	0.21	105	27 ±1.6	12 (11)	NR	NR	NR	NR	NR
High	1.0	20	27 ± 1.6	3 (15)	NR	NR	NR	NR	NR
**Rabi Enrolment 2004–2009 population based, Canada** **(****55**)									
Low	0.21–0.4	1244	26 (25,27)^∧∧^	251 (21)	512 (51)	525 (80)	132 (11)	166 (22)	288 (26)
High	1.0	1082	26 (25,27)^∧∧^	192 (18)	458 (51)	630 (90)	96 (9)	165 (24)	215 (23)
**Soraisham Enrolment 2010–2011 Population based, Canada** **(****56**)									
Low	0.21	445	26.3 ± 1.4	68	181 (47)	248 (56)	43 (10)	51 (16)	59 (13)
Intermediate	0.22–0.99	483	26.3 ± 1.3	72	179 (43)	259 (54)	45 (9)	36 (12)	50 (10)
High	1.0	581	25.8 ± 1.5	124	258 (55)^∧^	365 (63)^∧^	52 (9)	77 (19)	88 (15)
**Kapadia 2013, 2017 Enrolment 2009–2012, institutional, USA** **(****57**)									
Low	0.21	89	26 ± 1	17 (19)	14 (19)^∧^	27 (30%)	7 (8%)	4 (4%)	10 (11%)
High	1.0	110	26 ± 1	21 (19)	36 (40)	46 (42%)	7 (6%)	14 (13%)	21 (19%)

Death is defined as death before hospital discharge; NR, not reported; BPD, bronchopulmonary dysplasia—need for oxygen/respiratory support at 36 weeks corrected gestation; PDA, patent ductus arteriosus—need for medical and/or surgical treatment; NEC, necrotising enterocolitis, >Bell stage 3; ROP, retinopathy of prematurity, >grade 2 and/or plus disease; IVH, intraventricular hemorrhage, >grade 2

** *percentage calculated for survivors*,

# *percentage calculated for all infants*.

∧ *Data expressed as mean ± standard deviation, n (%), median (range)*.

∧∧ *Median (interquartile range)*.

*^∧^p < 0.05*.

The cohort studies (36, 55–57), nevertheless, suggest that the skills needed for oxygen blending and SpO_2_ titration may improve with time, possibly with better outcomes for the infants. These studies compared outcomes after changes to institutional and national delivery oxygen policies. Dawson et al. (36) compared the outcomes of 106 infants below 30 weeks gestation who were resuscitated with air after a change to institutional policy in 2006, to 20 historical cohorts that were resuscitated with 100% oxygen. This noted that 92% of air infants required supplemental oxygen by 5 min of age and that oxygen titration resulted in similar SpO_2_ course to “normal” term and preterm infants. Kapadia et al. compared the outcomes of 110 infants below 28 weeks gestation that were resuscitated with 100% oxygen titrated to target SpO_2_ 85–94% to 89 infants resuscitated with initial 21% oxygen, titrated to meet recommended guideline SpO_2_ after a change in institutional policy in 2011. No difference in mortality was noted but low oxygen infants had decreased risk of BPD (aOR 0.4, 95% confidence intervals: 0.2–0.9) and higher motor scores on the Bayley Scales of Infants and Toddler Assessment (57).

In Canada, Rabi et al. noted a higher risk of severe neurological injury or death (aOR 1.36. 95% CI: 1.11–1.66) in preterm infants born between 2004 and 2009 after a change in national resuscitation policy from using 100% oxygen to lower oxygen strategies (55). In a later cohort born between 2010 and 2011, Soraisham et al. (56) showed decreased risk of death and/or disability for infants receiving room air (*n* = 445) or intermediate (22–99%, *n* = 483) oxygen compared to those resuscitated with 100% oxygen (*n* = 581).

### Starting FiO_2_ for Preterm Infants: the Loss of Equipoise for Higher Oxygen Strategies

In a survey of 630 clinicians from 25 countries in 2015, almost all (>80%) would initiate resuscitation for preterm infants below 29 weeks gestation with FiO_2_ below 0.4. The most commonly used starting FiO_2_ was 0.3–0.4. Almost none would use FiO_2_ above 0.6 and only four respondents would use pure oxygen as they were limited by equipment availability (58). The lack of equipoise toward the use of high initial FiO_2_ is demonstrated by the difficulty in recruitment for the To2rpido study (54) which compared air to FiO_2_ 1.0 for resuscitation of infants below 32 weeks gestation.

The RCTs have examined various levels of initial FiO_2_, ranging from FiO_2_ 0.21–0.3 for low arms and FiO_2_ 0.6–1.0 for high arms. All of these studies titrated FiO_2_ to different target SpO_2_ levels and none compared oxygen titration to the previous gold standard of care: pure oxygen. Importantly, none have examined the FiO_2_ levels used most commonly by clinicians: 0.31–0.4 (58) and there are no studies examining impact of initial FiO_2_ on non-asphyxiated infants between 32 and 36 weeks gestation.

Importantly, none of the studies were powered sufficiently to examine either survival alone or survival without neurodevelopmental injury. To amalgamate existing data, three meta-analyses have now been conducted. Lui et al. identified 10 studies that randomized 914 infants to initial with FiO_2_ <0.4 or ≥0.4. Subgroup analyses were conducted for different FiO_2_ strata (0.21 vs. ≥ 0.4 to <0.6; 0.21 vs. ≥ 0.6 to 1.0; and ≥ 0.3 to <0.4 vs. ≥ 0.6 to 1.0 and found no difference in the primary outcomes of death and or disability between lower and higher oxygen strategies (59). Welsford et al. included cohort (*n* = 4) as well as RCTs (*n* = 10), totalling 5,697 patients ≤ 35 weeks gestation and again, found no difference in the risk of short-term mortality (*n* = 968, risk ratio (RR) 0.83, 95% confidence interval (CI) 0.50–1.37), long term mortality and neurodevelopmental outcomes (60). Oei et al. (61) examined individual patient data for infants <29 weeks gestation from 8 studies (*n* = 504, 37–39, 50–54) and again, found no difference in the risk of hospital death, bronchopulmonary dysplasia (BPD), severe intraventricular hemorrhage (IVH), or retinopathy of prematurity (ROP). See Table 2 for a summary of meta-analyses.

**Table 2:** Summary of meta-analyses for the use of oxygen in the delivery room stabilization of preterm infants.

	**Studies *n***	**Infants *n***	**Type**	**Comparator**	**Gestation (weeks)**	**Death-short term**	**BPD**	**IVH**	**NEC**	**ROP**	**Disability at 2 years**
Oei et al. (61)	8 RCT	504	IPD	FiO_2_ ≤ 0.3 vs. ≥ 0.6	<29	0.99 (0.52–1.91)	0.88 (0.68–1.14)	0.81 (0.52–1.27)	1.61 (0.77–3.36)	0.82 (0.46–1.46)	NR
Lui et al. (59)	10	914	Pooled	FiO_2_ < /≥ 0.4	<32	1.05 (0.68–1.63)	0.91 (0.72–1.14)	0.93 (0.51–1.71)	0.98 (0.51–1.87)	0.57 (0.24–1.36)	0.82 (0.49–1.35) 2 studies, *n* = 208
Oei et al.^#^ (62)	8	706	IPD	SpO_2_ <80% vs. >85%	<29	**2.1 (1.1**–**3.9)**^∧^	1.2 (0.8–1.8)	**4.7 (2.1**–**10.2)**^∧^	NR	1.6 (0.8–3.1)	NR
Welsford et al. (60)	10 RCT 4 cohorts	5,697^**^	Pooled	FiO_2_ “lower” vs. “higher”^*^	<35	0.83 (050–1.37) *N* = 968	(0.71–1.40) *N* = 843	0.96 (0.61–1.51) *N* = 795	1.34 (0.62–2.84) *N* = 847	0.73 (0.42–1.27) *N* = 806	1.14 (0.78–1.67) *N* = 389
					<28	0.92 (0.43–1.94) *N* = 467	0.90 (0.64–1.28) *N* = 467	0.84 (0.50–1.40) *N* = 441	1.62 (0.66–3.99) *N* = 441	0.75 (0.43–1.33) *N* = 441	1.08 (0.58–2.03) 1 study, *N* = 69
Oei et al.^#^ (63)	3 RCT	543 eligible	IPD	FiO_2_ ≤ 0.3 vs. ≥ 0.6 *N* = 539	<32	NR	NR	NR	NR	NR	Cognitive score <85: 0.8 (0.4–1.5) Any disability: 1.0 (0.8–1.3)
				SpO_2_ <80% vs. ≥80% *N* = 473	3 <32	NR	NR	NR	NR	NR	Cognitive score <85: **0.4 (0.2**–**0.8)**^∧^ Any disability: **0.6 (0.5**–**0.8)**^∧^

IPD, Individual Patient Data; BPD, bronchopulmonary dysplasia; IVH, Intraventricular hemorrhage, grades >3; NEC, necrotizing enterocolitis; ROP, retinopathy of prematurity;

* *exact FiO_2_ undefined*,

** *outcomes reported for RCTs only*,

∧ *p < 0.05*.

*Data expressed as Risk Ratio (95% Confidence Intervals) except for # (Odds Ratio, 95% Confidence Intervals)*.

### SpO_2_ Targeting: the Other Part of the Oxygen Question and Can It Be Achieved?

The lack of difference noted with initial FiO_2_ may be due to the way in which oxygen is titrated during stabilization. In Oei's meta-analysis, blinded studies, where oxygen was titrated by the research team without clinician input, had lower mortality rates in lower oxygen arms [RR 0.46, 95% Ci 0.23–0.92, *p* = 0.03; (61)]. The Canadian population studies (55, 56) noted a change in outcomes between two time periods in infants resuscitated with lower oxygen strategies and the necessary skills to titrate oxygen in response to SpO_2_ changes undoubtedly require experience (64). In experienced hands, SpO_2_ readings can be obtained even in very small infants by 2 min of age (65) but frequent manipulations may be necessary to achieve target SpO_2_ levels. SpO_2_ targeting is technically difficult, even within the nursery. Lim et al. analyzed 4,034 h of data from 45 infants in a neonatal intensive care unit (median gestation 30 weeks, IQR 27–32) and found that hyperoxia was directly related to the number of patients managed by the nurse. Infants were within target SpO_2_ ranges only 31% of the time (median, IQR 19–39%) and experienced a median of 25 FiO_2_ adjustments (range 16–41) each day (66).

Within the delivery room, SpO_2_ targeting could even be more technically challenging. In an observational study of 78 infants (median 27 weeks gestation), Goos et al. noted large deviations above [median (IQR)] of 4.4% SpO2 (1.4–6.5), and below target (8.2% (2.8–16.0) SpO_2_ in the delivery room. After the first 10 min, SpO2 levels were, respectively, above and below the limit for 11% (0–27) and 8% (0–23) of the time (67). In bench tests, Dekker et al. noted that the median (IQR) time required to achieved necessary FiO_2_ was 34.2 (21.8–69.1) s. During stabilization of preterm infants (median gestation 29 weeks), almost half (49%) of titrations were adjusted prior to achieving desired FiO_2_ levels (68). In a prospective observational study of 27 preterm infants (mean 28 weeks gestation, 962 g birthweight), White et al. found that infants spent almost two-thirds of the first 10 min of life with SpO_2_ outside target ranges [below by 28%, within by 35% and above by 37% of the time, (69)].

### “Normal” SpO_2_ for the Preterm Infant and Its Implications

Uncertainty regarding “normal SpO_2_” for a preterm infant requiring stabilization and respiratory support in the delivery room is illustrated by the results of a survey of 45 international CPGs. Of these, 36 had gestation specific recommendations, five did not provide SpO_2_ recommendations and 5-min SpO_2_ targets differed by up to 20% (70–90%). The most common recommendation was to adjust FiO_2_ to target 5-min SpO_2_ of 80–85% (70).

However, the consequences of reaching, not reaching or exceeding recommended SpO_2_ levels are unknown, not only for the preterm infants but also for term infants. The existing RCTs were designed to assess initial FiO_2_ only rather than specific SpO_2_ targets, which varied considerably from study to study. In an individual patient data analysis of 768 infants below 32 weeks gestation randomized to either lower (FiO_2_ ≤ 0.3) or higher (FiO_2_ ≥ 0.6) initial oxygen at delivery, infants that did not reach a minimum of SpO_2_ 80% by 5 min were more likely to have lower heart rates (mean difference −8.37 bpm, 95% CI −15.73 to −1.01), develop severe (grade III/IV) IVH (OR 2.4, 95% CI: 1.01–4.11) and to die. The risk of death increased with time taken to reach a minimum SpO_2_ of 80% and infants were less likely to reach SpO_2_ 80% by 5 min if respiratory support was initiated with less oxygen [FiO_2_ 0.3 vs. 0.6, OR 2.63, 95% CI: 1.21–5.74; (62)].

The relevance of SpO_2_ on preterm infant outcomes needs further evaluation. In a recent secondary analysis of 284 infants <32 weeks gestation that were enrolled in several delivery room trials, Katheria et al. showed that infants who did not reach a minimum SpO_2_ 80% by 5 min (*n* = 100, mean gestation 27.4 weeks) were more likely to die (16 vs. 4%), develop severe IVH (24 vs. 10%), have lower heart rates, require higher mean airway pressures and were given more oxygen compared to infants with higher SpO_2_ (71).

### The Association Between SpO_2_ at Birth and Cerebral Oxygenation

There is now emerging evidence that longer-term outcomes may be impacted by SpO_2_ at birth. Both hypoxia and hyperoxia causes rapid cellular injury and compromise. In the early 1990s, Lundstrom et al. noted decreased cerebral blood flow in preterm infants resuscitated with higher initial FiO_2_ (0.8) compared to air but as noted before, the implications of this finding were unclear as all infants survived delivery room resuscitation (35). The advent of newer technologies, such as Near Infrared Spectroscopy (NIRS) show that regional cerebral oxygen saturations (rcSO_2_) are exquisitely sensitive to changes in FiO_2_ even in the first few minutes of life (72). Kenosi et al. measured rcSO_2_ in 47 preterm infants (mean gestation 29.4 weeks) who were all initially given FiO_2_ 0.3. These infants were then divided into two groups according to their subsequent FiO_2_ needs ( ≤ or ≥0.3). Those needing ≥ FiO_2_ 0.3 showed evidence of increased cerebral hypoxia (rsSO_2_ <55%) but no difference in the degree of cerebral hyperoxia, suggesting that infants with SpO_2_ may need more rapid upward FiO_2_ titration to prevent cerebral hypoxia (73). The COSGOD III study aims to recruit 726 infants <32 weeks gestation to determine if cerebral NIRS measurements can influence the risk of survival and cerebral injury (74). Using rcSO_2_ measurements as well as SpO_2_ may serve to further inform on optimum oxygen needs for the transitioning preterm infant.

### The Impact of Oxygenation at Birth and Long-Term Outcomes

No study has compared the current practice of using oxygen titration strategies to the previous standard treatment of using only FiO_2_ 1.0. The follow-up cohorts from 2 RCTs: Boronat et al., who examined 206 children below 32 weeks gestation randomized to initial FiO_2_ 0.3 or 0.6 (75) and Thamin et al., who examined 238 infants below 32 weeks gestation randomized to resuscitation with either FiO_2_ 1.0 or 0.21 (76), found no difference in the major outcomes of death or disability with different initial FiO_2_. However, meta-analysis of these two studies with individual patient data found significantly lower mean cognitive scores in males ≥ 29 weeks gestation initially given FiO_2_ ≤ 0.3 compared to those given FiO_2_ ≥ 0.6. In addition, infants with 5-min SpO_2_ ≥ 80% were significantly less like to be disabled/deceased (OR 0.43, 95% CI: 0.27–0.67) or in survivors, be disabled [OR: 0.57 (0.36–0.89)], compared to those with 5 min SpO_2_ <80% (63).

### The Way Forward: Individualization May Be the Key

Perhaps the amount of oxygenation needed by preterm infants needs to be individualized. The To_2_rpido study, for example, showed that mortality rates of infants ≥29 weeks gestation were not influenced by initial FiO_2_, in contrast to infants below 29 weeks who were more likely die if exposed initially to air (54). More mature infants were more likely to overshoot recommended SpO_2_ targets despite FiO_2_ adjustment if supported was started with higher levels of oxygen (54). The consequences of “overshooting” SpO_2_ targets still remain unclear. In the meta-analysis of long-term outcomes of RCTs, mature female infants (≥29 weeks gestation) who had respiratory support initiated with higher FiO_2_ levels (≥0.6) had higher cognitive scores than all other infants, especially male infants >29 weeks gestation who were given lower initial FiO_2_ (≤0.3) (63). Indeed, this could reflect better lung maturity in females. Observations from 102 infants (median gestation 29 weeks) found significantly higher SpO_2_ in females in the first 10 min compared to males (77), suggesting that males and very preterm infants may need more oxygen after birth, either by starting on a higher initial FiO_2_ or with more rapid oxygen titration strategies.

### The Major Knowledge Gaps for Delivery Room Oxygenation of the Preterm Infant

**Figure 1:**
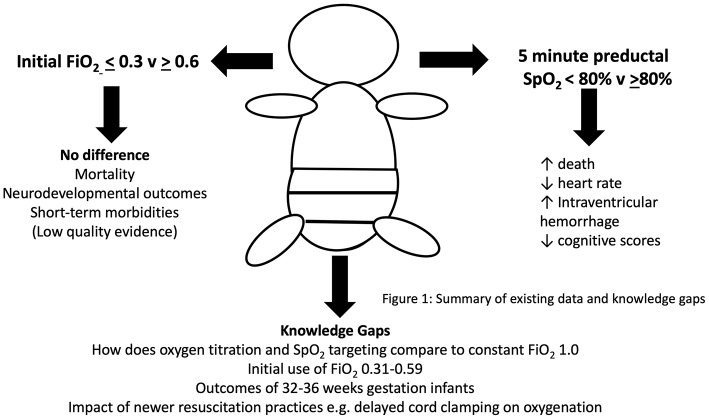
Current evidence and knowledge gaps for the use of oxygen in preterm newborn resuscitation.

Currently, optimum oxygenation during the first 10 min of life in preterm infants is unknown but data show that oxygen levels, even within the few first minutes, have enormous potential to influence death and longer-term outcomes. However, there are significant deficits to current knowledge that need to be address before contemporary practice can be considered safe (see Figure 1).

For example, research must be conducted to address the needs of more mature preterm infants (32–36 weeks gestation) that are the largest global population of preterm infants (>80%). This group of infants are at significant risk of neurodevelopmental issues when compared to full-term infants even though most are considered physically healthy (78). Whether delivery room oxygen strategies have the potential to change their long-term outcomes needs to be determined. Furthermore, the feasibility of oxygen titration strategies in under-resourced countries, which also carry the global burden of prematurity, is unknown. Equipment needed to blend and monitor oxygen use is expensive and clinicians in these countries may be restricted to using either only air or pure oxygen if these are unavailable (79).

Finally, the implications of evolving resuscitation practices on the oxygen needs of the preterm infant need to be evaluated. The clinical trials have so far, not addressed the impact of delivery room practices such as delayed cord clamping (80), less invasive surfactant administration (81), and increasing use of non-invasive respiratory support. Animal and physiological studies of human infants show that these practices may have considerable impact on oxygenation status. For example, glottic opening is enhanced higher (e.g., FiO_2_ 1.0) oxygen exposure than air, which may then impact on the respiratory status of unintubated infants (82).

## Conclusions and Clinical Summary

In conclusion, current recommendations for the use of oxygen during delivery room stabilization of preterm infants at birth are determined primarily from data that are amalgamated from term infants and older resuscitation strategies. These significant knowledge gaps are acknowledged by expert committees. Even though great importance is placed on initial FiO_2_ and the need to avoid hyperoxia, further evaluation of other aspects of oxygen handling, e.g., SpO_2_ targeting is needed. Individualization of oxygen strategies also appears necessary, with some infants e.g., males and very preterm infants, requiring more oxygen to prevent hypoxia than females, and older preterm infants. The implications of current recommendations (oxygen targeting) for lower resource countries without access to blending and monitoring equipment, also need to be considered. Further evidence for best practice is needed from large scale, RCTs to determine not only the short-term but also the long-term implications of this practice (83).

## Author Contributions

JO developed the manuscript and approved the final version for submission. MV revised and approved the final manuscript for submission.

### Conflict of Interest Statement

The authors declare that the research was conducted in the absence of any commercial or financial relationships that could be construed as a potential conflict of interest.

## References

[1] Obladen M. History of neonatal resuscitation. Part 2: oxygen and other drugs. Neonatology. (2009) 95:91–6. 10.1159/000151761 18787343

[2] Klaus M Meyer BP. Oxygen therapy for the newborn. Pediatr Clin North Am. (1966) 13:731–52. 10.1016/S0031-3955(16)31880-6 5946302

[3] Roberts MH. Emergencies encountered in the neonatal period. J Am Med Assoc. (1949) 139:439–44. 10.1001/jama.1949.02900240017004 18107959

[4] Apgar V. The first twelve minutes. Trans N Engl Obstet Gynecol Soc. (1957) 11:39–47. 13556842

[5] Apgar V Girdany BR McIntosh R Taylor HC Jr. Neonatal anoxia. I. A study of the relation of oxygenation at birth to intellectual development. Pediatrics. (1955) 15:653–62. 14384365

[6] Ramji S Ahuja S Thirupuram S Rootwelt T Rooth G Saugstad OD Resuscitation of asphyxic newborn infants with room air or 100% oxygen. Pediatr Res. (1993) 34:809–12. 10.1203/00006450-199312000-00023 8108199

[7] Saugstad OD Rootwelt T Aalen O. Resuscitation of asphyxiated newborn infants with room air or oxygen: an international controlled trial: the Resair 2 study. Pediatrics. (1998) 102:e1. 10.1542/peds.102.1.e1 9651453

[8] Ramji S Rasaily R Mishra PK Narang A Jayam S Kapoor AN. Resuscitation of asphyxiated newborns with room air or 100% oxygen at birth: a multicentric clinical trial. Indian Pediatr. (2003) 40:510–7. 12824660

[9] Vento M Asensi M Sastre J García-Sala F Pallardó FV Viña J. Resuscitation with room air instead of 100% oxygen prevents oxidative stress in moderately asphyxiated term neonates. Pediatrics. (2001) 107:642–7. 10.1542/peds.107.4.642 11335737

[10] Vento M Asensi M Sastre J Lloret A García-Sala F Viña J. Oxidative stress in asphyxiated term infants resuscitated with 100% oxygen. J Pediatr. (2003) 142:240–6. 10.1067/mpd.2003.91 12640369

[11] Vento M Sastre J Asensi MA Viña J. Room-air resuscitation causes less damage to heart and kidney than 100% oxygen. Am J Respir Crit Care Med. (2005) 172:1393–8. 10.1164/rccm.200412-1740OC 16141440

[12] Bajaj N Udani RH Nanavati RN. Room air vs. 100 per cent oxygen for neonatal resuscitation: a controlled clinical trial. J Trop Pediatr. (2005) 51:206–11. 10.1093/tropej/fmh086 15927951

[13] Toma AI Nanea M Scheiner M Mitu R Petrescu I Matu E Efectele gazului folosit pentru reanimarea nou- nascutului asupra hemodinamicii post- resuscitare [Effects of the gas used in the resuscitation of the newborn in the post-resuscitation haemodynamics]. Asfixia Perinat. (2006) 33–34.

[14] Recommendations on resuscitation of babies at birth International Liaison Committee on Resuscitation. Resuscitation. (1998) 37:103–10. 9671085 10.1016/s0300-9572(98)00041-0

[15] International Liaison Committee on Resuscitation The International Liaison Committee on Resuscitation (ILCOR) consensus on science with treatment recommendations for pediatric and neonatal patients: pediatric basic and advanced life support. Pediatrics. (2006) 117:e955–77. 10.1542/peds.2006-0206 16618790

[16] Wyllie J Perlman JM Kattwinkel J Atkins DL Chameides L Goldsmith JP. Part 11: neonatal resuscitation: 2010 International consensus on cardiopulmonary resuscitation and emergency cardiovascular care science with treatment recommendations. Resuscitation. (2010) 81(Suppl. 1):e260–87. 10.1161/CIRCULATIONAHA.110.971127 20956039

[17] Mariani G Dik PB Ezquer A Aguirre A Esteban ML Perez C. Pre-ductal and post-ductal O_2_ saturation in healthy term neonates after birth. J Pediatr. (2007) 150:418–21. 10.1016/j.jpeds.2006.12.015 17382123

[18] Dawson JA Kamlin CO Vento M Wong C Cole TJ Donath SM. Defining the reference range for oxygen saturation for infants after birth. Pediatrics. (2010) 125:e1340–7. 10.1542/peds.2009-1510 20439604

[19] Wyckoff MH Aziz K Escobedo MB Kapadia VS Kattwinkel J Perlman JM. Part 13: neonatal resuscitation: 2015 American Heart Association Guidelines update for cardiopulmonary resuscitation and emergency cardiovascular care. Circulation. (2015) 132(18 Suppl. 2):S543–60. 10.1161/CIR.0000000000000267 26473001

[20] Novak CM Ozen M Burd I. Perinatal brain injury: mechanisms, prevention, and outcomes. Clin Perinatol. (2018) 45:357–75. 10.1016/j.clp.2018.01.015 29747893

[21] Saugstad OD Sanderud J. Circulatory effects of oxygen radicals. Biomed Biochim Acta. (1989) 48:S20–4 2730608

[22] Saugstad OD Aasen AO. Plasma hypoxanthine concentrations in pigs. A prognostic aid in hypoxia. Eur Surg Res. (1980) 12:1. 10.1159/000128117 7408920

[23] Tan A Schulze A O'Donnell CP Davis PG Air versus oxygen for resuscitation of infants at birth. Cochrane Database Syst Rev. (2005) 18:CD002273 10.1002/14651858.CD002273.pub3 PMC7017642 15846632

[24] Davis PG Tan A O'Donnell CP Schulze A Resuscitation of newborn infants with 100% oxygen or air: a systematic review and meta-analysis. Lancet. (2004) 364:1329–33. 10.1016/S0140-6736(04)17189-4 15474135

[25] Saugstad OD Ramji S Vento M. Resuscitation of depressed newborn infants with ambient air or pure oxygen: a meta-analysis. Biol Neonate. (2005) 87:27–34. 10.1159/000080950 15452400

[26] Rabi Y Rabi D Yee W. Room air resuscitation of the depressed newborn: a systematic review and meta-analysis. Resuscitation. (2007) 72:353–63. 10.1016/j.resuscitation.2006.06.134 17240032

[27] Zhu JJ Wu MY Which is better to resuscitate asphyxiated newborn infants: room air or pure oxygen? Zhonghua Er Ke Za Zhi. (2007) 45:644–9. 18021551

[28] Saugstad OD Ramji S Soll RF Vento M. Resuscitation of newborn infants with 21% or 100% oxygen: an updated systematic review and meta-analysis. Neonatology. (2008) 94:176–82. 10.1159/000143397 18612215

[29] Guay J Lachapelle J. No evidence for superiority of air or oxygen for neonatal resuscitation: a meta-analysis. Can J Anaesth. (2011) 58:1075–82. 10.1007/s12630-011-9589-0 21971742

[30] Welsford M Nishiyama C Shortt C Isayama T Dawson JA Weiner G. Room air for initiating term newborn resuscitation: a systematic review with meta-analysis. Pediatrics. (2019) 143:e20181825. 10.1542/peds.2018-1825 30578325

[31] Saugstad OD Vento M Ramji S Howard D Soll RF Neurodevelopmental outcome of infants resuscitated with air or 100% oxygen: a systematic review and meta-analysis. Neonatology. (2012) 102:98–103. 10.1159/000333346 22677698

[32] Toth B Becker A Seelbach-Göbel B. Oxygen saturation in healthy newborn infants immediately after birth measured by pulse oximetry. Arch Gynecol Obstet. (2002) 266:105–7. 10.1007/s00404-001-0272-5 12049291

[33] Kamlin CO O'Donnell CP Davis PG Morley CJ. Oxygen saturation in healthy infants immediately after birth. J Pediatr. (2006) 148:585–9. 10.1016/j.jpeds.2005.12.050 16737865

[34] Rabi Y Yee W Chen SY Singhal N. Oxygen saturation trends immediately after birth. J Pediatr. (2006) 148:590–4. 10.1016/j.jpeds.2005.12.047 16737866

[35] Lundstrøm KE Pryds O Greisen G. Oxygen at birth and prolonged cerebral vasoconstriction in preterm infants. Arch Dis Childhood. (1995) 73:F81–6. 10.1136/fn.73.2.F81 7583611 PMC2528504

[36] Dawson JA Kamlin CO Wong C te Pas AB O'Donnell CP Donath SM Oxygen saturation and heart rate during delivery room resuscitation of infants <30 weeks' gestation with air or 100% oxygen. Arch Dis Child Fetal Neonatal Ed. (2009) 94:F87–91. 10.1136/adc.2008.141341 18703572

[37] Wang CL Anderson C Leone TA Rich W Govindaswami B Finer NN Resuscitation of preterm neonates by using room air or 100% oxygen. Pediatrics. (2008) 121:1083–9. 10.1542/peds.2007-1460 18519476

[38] Vento M Moro M Escrig R Arruza L Villar G Izquierdo I. Preterm resuscitation with low oxygen causes less oxidative stress, inflammation, and chronic lung disease. Pediatrics. (2009) 124:e439–49. 10.1542/peds.2009-0434 19661049

[39] Escrig R Arruza L Izquierdo I Villar G Sáenz P Gimeno A. Achievement of targeted saturation values in extremely low gestational age neonates resuscitated with low or high oxygen concentrations: a prospective, randomized trial. Pediatrics. (2008) 121:875–81. 10.1542/peds.2007-1984 18450889

[40] Liggins GC Kitterman JA. Maturation of the fetal lung. Dan Med Bull. (1979) 26:129–30. 446111

[41] Sardesai S Biniwale M Wertheimer F Garingo A Ramanathan R. Evolution of surfactant therapy for respiratory distress syndrome: past, present, and future. Pediatr Res. (2017) 81:240–8. 10.1038/pr.2016.203 27706130

[42] Frank L Price LT Whitney PL. Possible mechanism for late gestational development of the antioxidant enzymes in the fetal rat lung. Biol Neonate. (1996) 70:116–26. 10.1159/000244356 8864431

[43] Higgins RD. Oxygen saturation and retinopathy of prematurity. Clin Perinatol. (2019) 46:593–9. 10.1016/j.clp.2019.05.008 31345549

[44] Northway WH Jr Rosan RC Porter DY. Pulmonary disease following respirator therapy of hyaline-membrane disease. Bronchopulmonary dysplasia. N Engl J Med. (1967) 276:357–68. 10.1056/NEJM196702162760701 5334613

[45] Askie LM Darlow BA Finer N Schmidt B Stenson B Tarnow-Mordi W. Association between oxygen saturation targeting and death or disability in extremely preterm infants in the neonatal oxygenation prospective meta-analysis collaboration. JAMA. (2018) 319:2190–201. 10.1001/jama.2018.5725 29872859 PMC6583054

[46] Svenningsen NW Stjernqvist K Stavenow S Hellström-Westas L. Neonatal outcome of extremely small low birthweight liveborn infants below 901 g in a Swedish population. Acta Paediatr Scand. (1989) 78:180–8. 10.1111/j.1651-2227.1989.tb11054.x 2929341

[47] Grave GD Kennedy C Jehle J Sokoloff L. The effects of hyperoxia on cerebral blood flow in newborn dogs. Neurology. (1970) 20:397–8. 10.1212/WNL.20.6.613 5535033

[48] Harling AE Beresford MW Vince GS Bates M Yoxall CW. Does the use of 50% oxygen at birth in preterm infants reduce lung injury? Arch Dis Child Fetal Neonatal Ed. (2005) 90:F401–5. 10.1136/adc.2004.059287 15863491 PMC1721933

[49] Armanian AM Badiee Z. Resuscitation of preterm newborns with low concentration oxygen versus high concentration oxygen. J Res Pharm Pract. (2012) 1:25–9. 10.4103/2279-042X.99674 24991584 PMC4076850

[50] Rabi Y Singhal N Nettel-Aguirre A. Room-air versus oxygen administration for resuscitation of preterm infants: the ROAR study. Pediatrics. (2011) 128:e374–81. 10.1542/peds.2010-3130 21746729

[51] Kapadia VS Chalak LF Sparks JE Allen JR Savani RC Wyckoff MH. Resuscitation of preterm neonates with limited versus high oxygen strategy. Pediatrics. (2013) 132:1488–96. 10.1542/peds.2013-0978 24218465 PMC3838529

[52] Aguar M Izquierdo M Brugada M Preterm babies randomly assigned to be blindly resuscitated with higher (60%) vs. lower (30%) initial FIO_2_: effects on oxidative stress and mortality. EPAS. (2014).

[53] Rook D Schierbeek H Vento M Vlaardingerbroek H van der Eijk AC Longini M. Resuscitation of preterm infants with different inspired oxygen fractions. J Pediatr. (2014) 164:1322–6 e3. 10.1016/j.jpeds.2014.02.019 24655537

[54] Oei JL Saugstad OD Lui K Wright IM Smyth JP Craven P. Targeted oxygen in the resuscitation of preterm infants, a randomized clinical trial. Pediatrics. (2017) 139:e20161452. 10.1542/peds.2016-1452 28034908

[55] Rabi Y Lodha A Soraisham A Singhal N Barrington K Shah PS. Outcomes of preterm infants following the introduction of room air resuscitation. Resuscitation. (2015) 96:252–9. 10.1016/j.resuscitation.2015.08.012 26359156

[56] Soraisham AS Rabi Y Shah PS Singhal N Synnes A Yang J. Neurodevelopmental outcomes of preterm infants resuscitated with different oxygen concentration at birth. J Perinatol. (2017) 37:1141–7. 10.1038/jp.2017.83 28594395

[57] Kapadia VS Lal CV Kakkilaya V Heyne R Savani RC Wyckoff MH. Impact of the neonatal resuscitation program-recommended low oxygen strategy on outcomes of infants born preterm. J Pediatr. (2017) 191:35–41. 10.1016/j.jpeds.2017.08.074 29173319 PMC5726565

[58] Oei JL Ghadge A Coates E Wright IM Saugstad OD Vento M. Clinicians in 25 countries prefer to use lower levels of oxygen to resuscitate preterm infants at birth. Acta Paediatr. (2016) 105:1061–6. 10.1111/apa.13485 27228325

[59] Lui K Jones LJ Foster JP Davis PG Ching SK Oei JL. Lower versus higher oxygen concentrations titrated to target oxygen saturations during resuscitation of preterm infants at birth. Cochrane Database Syst Rev. (2018) 5:CD010239. 10.1002/14651858.CD010239.pub2 29726010 PMC6494481

[60] Welsford M Nishiyama C Shortt C Weiner G Roehr CC Isayama T. Initial oxygen use for preterm newborn resuscitation: a systematic review with meta-analysis. Pediatrics. (2018) 143:e20181828. 10.1542/peds.2018-1828 30578326

[61] Oei JL Vento M Rabi Y Wright I Finer N Rich W. Higher or lower oxygen for delivery room resuscitation of preterm infants below 28 completed weeks gestation: a meta-analysis. Arch Dis Child Fetal Neonatal Ed. (2017) 102:F24–30. 10.1136/archdischild-2016-310435 27150977

[62] Oei JL Finer NN Saugstad OD Wright IM Rabi Y Tarnow-Mordi W. Outcomes of oxygen saturation targeting during delivery room stabilisation of preterm infants. Arch Dis Child Fetal Neonatal Ed. (2018) 103:F446–54. 10.1136/archdischild-2016-312366 28988158 PMC6490957

[63] Oei JL Kapadia V Rabi Y Saugstad OD Rook D Vermeulen MJ Neurodevelopmental outcomes of preterm infants after randomisation to initial resuscitation with lower (FiO_2_ <0.3) or higher (FiO_2_ > 0.6) initial oxygen levels, an individual patient meta-analysis. J Pediatr. (2019). 10.1136/archdischild-2021-321565 34725105

[64] van Zanten HA Pauws SC Beks EC Stenson BJ Lopriore E Te Pas AB. Improving manual oxygen titration in preterm infants by training and guideline implementation. Eur J Pediatr. (2017) 176:99–107. 10.1007/s00431-016-2811-x 27888413 PMC5219007

[65] Gandhi B Rich W Finer N. Time to achieve stable pulse oximetry values in VLBW infants in the delivery room. Resuscitation. (2013) 84:970–3. 10.1016/j.resuscitation.2012.12.007 23238422

[66] Lim K Wheeler KI Gale TJ Jackson HD Kihlstrand JF Sand C. Oxygen saturation targeting in preterm infants receiving continuous positive airway pressure. J Pediatr. (2014) 164:730–6 e1. 10.1016/j.jpeds.2013.11.072 24433828

[67] Goos TG Rook D van der Eijk AC Kroon AA Pichler G Urlesberger B. Observing the resuscitation of very preterm infants: are we able to follow the oxygen saturation targets? Resuscitation. (2013) 84:1108–13. 10.1016/j.resuscitation.2013.01.025 23376585

[68] Dekker J Stenning FJ Willms LJFB Martherus T Hooper SB Te Pas AB. Time to achieve desired fraction of inspired oxygen using a T-piece ventilator during resuscitation of preterm infants at birth. Resuscitation. (2019) 136:100–4. 10.1016/j.resuscitation.2019.01.024 30708072

[69] White LN Thio M Owen LS Kamlin CO Sloss S Hooper SB. Achievement of saturation targets in preterm infants <32 weeks' gestational age in the delivery room. Arch Dis Child Fetal Neonatal Ed. (2017) 102:F423–7. 10.1136/archdischild-2015-310311 28302696

[70] Wilson A Vento M Shah PS Saugstad O Finer N Rich W. A review of international clinical practice guidelines for the use of oxygen in the delivery room resuscitation of preterm infants. Acta Paediatr. (2018) 107:20–7. 10.1111/apa.14012 28792628

[71] Katheria AC Hassen K Rich WD Finer N Resuscitation outcomes of infants that do not achieve target saturations, in Abstract. Pediatric Academic Societies Meeting. Baltimore, MD (2019). 10.1038/s41372-019-0491-x 31488904

[72] Pichler G Schmölzer GM Urlesberger B. Cerebral tissue oxygenation during immediate neonatal transition and resuscitation. Front Pediatr. (2017) 5:29. 10.3389/fped.2017.00029 28280719 PMC5322290

[73] Kenosi M O'Toole JM Livingston V Hawkes GA Boylan GB O'Halloran KD. Effects of fractional inspired oxygen on cerebral oxygenation in preterm infants following delivery. J Pediatr. (2015) 167:1007–12 e1. 10.1016/j.jpeds.2015.07.063 26387011

[74] Pichler G Baumgartner S Biermayr M Dempsey E Fuchs H Goos TG. Cerebral regional tissue Oxygen Saturation to Guide Oxygen Delivery in preterm neonates during immediate transition after birth (COSGOD III): an investigator-initiated, randomized, multi-center, multi-national, clinical trial on additional cerebral tissue oxygen saturation monitoring combined with defined treatment guidelines versus standard monitoring and treatment as usual in premature infants during immediate transition: study protocol for a randomized controlled trial. Trials. (2019) 20:178. 10.1186/s13063-019-3258-y 30894226 PMC6427901

[75] Boronat N Aguar M Rook D Iriondo M Brugada M Cernada M. Survival and neurodevelopmental outcomes of preterms resuscitated with different oxygen fractions. Pediatrics. (2016) 138:e20161405. 10.1542/peds.2016-1405 27940687

[76] Thamrin V Saugstad OD Tarnow-Mordi W Wang YA Lui K Wright IM Preterm infant outcomes after randomization to initial resuscitation with FiO_2_ 0.21 or 1.0. J Pediatr. (2018) 201:55–61 e1. 10.1016/j.jpeds.2018.05.053 30251639

[77] Vento M Cubells E Escobar JJ Escrig R Aguar M Brugada M. Oxygen saturation after birth in preterm infants treated with continuous positive airway pressure and air: assessment of gender differences and comparison with a published nomogram. Arch Dis Child Fetal Neonatal Ed. (2013) 98:F228–32. 10.1136/archdischild-2012-302369 23123635

[78] Cheong JL Doyle LW Burnett AC Lee KJ Walsh JM Potter CR. Association between moderate and late preterm birth and neurodevelopment and social-emotional development at age 2 years. JAMA Pediatr. (2017) 171:e164805. 10.1001/jamapediatrics.2016.4805 28152144

[79] Trevisanuto D Cavallin F Arnolda G Chien TD Lincetto O Xuan NM. Equipment for neonatal resuscitation in a middle-income country: a national survey in Vietnam. BMC Pediatr. (2016) 16:139. 10.1186/s12887-016-0664-0 27544219 PMC4992562

[80] Tarnow-Mordi W Morris J Kirby A Robledo K Askie L Brown R. Delayed versus immediate cord clamping in preterm infants. N Engl J Med. (2017) 377:2445–55. 10.1056/NEJMoa1711281 29081267

[81] Aldana-Aguirre JC Pinto M Featherstone RM Kumar M. Less invasive surfactant administration versus intubation for surfactant delivery in preterm infants with respiratory distress syndrome: a systematic review and meta-analysis. Arch Dis Child Fetal Neonatal Ed. (2017) 102:F17–23. 10.1136/archdischild-2015-310299 27852668

[82] Crawshaw JR Kitchen MJ Binder-Heschl C Thio M Wallace MJ Kerr LT. Laryngeal closure impedes non-invasive ventilation at birth. Arch Dis Child Fetal Neonatal Ed. (2018) 103:F112–9. 10.1136/archdischild-2017-312681 29054974 PMC5868244

[83] The To2rpido Study Targeted Oxygenation in the Resuscitation of Premature Infants and their Developmental Outcome. ACTRN12610001059055. The Australian New Zealand Clinical Trials Registry. Available online at: https://www.anzctr.org.au/Trial/Registration/TrialReview.aspx?id=335870&isClinicalTrial=False (accessed June 12, 2019).

